# Chickens treated with a nitric oxide inhibitor became more resistant to *Plasmodium gallinaceum* infection due to reduced anemia, thrombocytopenia and inflammation

**DOI:** 10.1186/1297-9716-44-8

**Published:** 2013-02-11

**Authors:** Barbarella Matos de Macchi, Farlen José Bebber Miranda, Fernanda Silva de Souza, Eulógio Carlos Queiroz de Carvalho, Antônio Peixoto Albernaz, José Luiz Martins do Nascimento, Renato Augusto DaMatta

**Affiliations:** 1 Laboratório de Biologia Celular e Tecidual, Centro de Biociências e Biotecnologia, Universidade Estadual do Norte Fluminense, 28013-602, Campos dos Goytacazes, RJ, Brazil; 2 Laboratório de Neuroquímica, Instituto de Ciências Biológicas, Universidade Federal do Pará, Av. Augusto Corrêa 1, 66075-110, Belém, PA, Brazil; 3 Laboratório de Morfologia e Patologia Animal, Centro de Ciências e Tecnologias Agropecuárias, Universidade Estadual do Norte Fluminense, 28013-602, Campos dos Goytacazes, RJ, Brazil; 4 Laboratório de Clínica e Cirurgia Animal, Centro de Ciências e Tecnologias Agropecuárias, Universidade Estadual do Norte Fluminense, 28013-602, Campos dos Goytacazes, RJ, Brazil

## Abstract

Malaria is a serious infectious disease caused by parasites of the *Plasmodium* genus that affect different vertebrate hosts. Severe malaria leads to host death and involves different pathophysiological phenomena such as anemia, thrombocytopenia and inflammation. Nitric oxide (NO) is an important effector molecule in this disease, but little is known about its role in avian malaria models. *Plasmodium gallinaceum-* infected chickens were treated with aminoguanidine (AG), an inhibitor of inducible nitric oxide synthase, to observe the role of NO in the pathogenesis of this avian model. AG increased the survival of chickens, but also induced higher parasitemia. Treated chickens demonstrated reduced anemia and thrombocytopenia. Moreover, erythrocytes at different stages of maturation, heterophils, monocytes and thrombocytes were infected by *Plasmodium gallinaceum* and animals presented a generalized leucopenia. Activated leukocytes and thrombocytes with elongated double nuclei were observed in chickens with higher parasitemia; however, eosinophils were not involved in the infection. AG reduced levels of hemozoin in the spleen and liver, indicating lower inflammation. Taken together, the results suggest that AG reduced anemia, thrombocytopenia and inflammation, explaining the greater survival rate of the treated chickens.

## Introduction

Malaria remains one of the most globally-important infectious diseases, particularly in terms of morbidity, mortality and deleterious economic consequences. This disease affects half a billion people worldwide every year and more than 2 million people die yearly from severe malaria [1]. This disease presents a number of different clinical symptoms, with a variety of pathological consequences associated with severe malaria, such as acute respiratory distress, renal failure, severe anemia and cerebral malaria, all of which can arise from infection with *Plasmodium falciparum*[1,2].

Several studies on the complex physiopathology of malaria have been published, but the cellular and molecular mechanisms involved in its pathogenesis remain unclear. Thus, experimental models have been established in order to address these issues and to develop better therapeutic procedures. Different malaria models with peculiar characteristics have been developed in monkeys, mice, rats and birds [3,4]. Investigations regarding avian hemoprotozoa have provided many fundamental discoveries about malaria, including the existence of the extra-erythrocytic stages occurring with *Plasmodium gallinaceum* infections [5-7]. *Plasmodium gallinaceum* (Apicomplexa: Haemospororida) infects chickens and was first described by Brumpt in 1935, constituting a versatile model for studying alternative treatments for human malaria [5,7,8]. This model dominated studies of the biology of the parasite and chemotherapeutic research from 1890 until 1940 [4,7]. Recently, this model has come back into use [4,8-14].

Several hypotheses exist to explain the mechanisms involved in malaria pathogenesis, such as anemia, thrombocytopenia and inflammation. One inflammatory mediator studied in malaria is nitric oxide (NO) [13,15-17]. NO is cytotoxic and cytostatic to blood stage malaria parasites in vitro [18-20], but the precise mechanism by which NO mediates an in vivo effect is not known. NO, when produced in large quantities by the inducible isoform of the NO synthase, has the potential to kill a variety of pathogens [21-23]. Whether NO controls, or not, acute parasitemia in malaria is unclear [24]. Increased production of endogenous NO during blood stage malaria has been correlated with protection against *P. chabaudi* infection in mice [18]. However, several studies show the dichotomous role of NO in malaria pathogenesis. Ghigo et al. demonstrated that neural changes in murine malaria increase cytokine levels and NO production by inducible NO synthase [15]. Evidence of this activation was demonstrated in the brain tissues of children with fatal malaria [17]. However, in the murine model, other authors have shown severe malaria in animals knocked out for inducible NO synthase [16]. In a previous study, we showed a positive correlation between NO production, by macrophages of chickens, with increased infection by *P. gallinaceum*[13]; however, little is known about the role of this radical in avian malaria.

Thrombocytopenia is also found in humans [25-27] and mice [28,29] malaria and was recently described in an avian malaria model in chickens that were experimentally infected with *P. juxtanucleare*[12], clearly indicating an important role for platelets/thrombocytes in the pathogenesis of this disease. Platelets are activated during the course of the malaria infection [26] and release microparticles that modulate the cytoadherence of parasitized erythrocytes on the brain endothelium [30,31], with ensuing platelet aggregation [30,32]. Thrombocytopenia is thought to occur as a consequence of platelet consumption and is probably part of the pathogenesis of malaria [26].

We have observed that macrophages of chickens infected with *P. gallinaceum* produce high amounts of NO [13]. As such, we evaluated the role of NO during the infection of chickens with *P. gallinaceum* by blocking NO production using aminoguanidine (AG) treatment. This treatment increased the survival of chickens during the acute phase of the infection, reduced anemia and thrombocytopenia, and lowered levels of hemozoin pigment in the spleen and liver. Moreover, blood lymphocytes, heterophils, monocytes and thrombocytes became activated and atypical thrombocytes were observed. These results suggest that the increased survival of AG- treated infected chickens was mediated by reduced anemia, thrombocytopenia and inflammation.

## Material and methods

### Parasite and chickens

The protozoan *P. gallinaceum*, strain 8A, was kindly provided by Dr Antoniana Krettli from the Centro de Pesquisa Renè Rachou, Fundação Oswaldo Cruz, Minas Gerais, Brazil. The parasite was maintained by successive passages in chickens. Hubbard chickens (1 day-old) were acquired from commercial establishments in Marechal Floriano, Espírito Santo, Brazil. Chickens were maintained in cages at the animal house of the Universidade Estadual do Norte Fluminense, Rio de Janeiro, Brazil, with water and a balanced feed (cocciodiostatic free), *ad libitum*. Chickens were infected on the 35^th^ day.

This study was carried out in strict accordance with the Brazilian Law #11794/08. The animal studies protocol was reviewed and approved by the Committee on the Ethics of Animal Experiments of the Universidade Estadual do Norte Fluminense (Permit Number: 100).

### Treatment with AG and infection with *Plasmodium gallinaceum*

Daily treatment with AG (25 mg/Kg) by the intraperitoneal route started two days before infection with the parasite. The dose was based on previous studies [33-35]. For infection, chickens were inoculated intramuscularly with 5 × 10^6^ erythrocytes parasitized by *P. gallinaceum*[13]. Chickens were clinically examined and monitored daily from the 4^th^ day post-infection (dpi); the assessment of the clinical manifestations was notated without score subdivision.

### Experimental groups and blood collection

Chickens were distributed randomly into experimental groups (G1: noninfected; G2: noninfected treated with AG; G3: infected untreated; G4: infected treated with AG). G1 and G2 had 5 chickens and G3 and G4 had 12 chickens. All chickens in G1 and G2 had their blood collected every four days after infection. G3 and G4 were divided into two subgroups of 6 chickens each. The subgroups were bled every four days, but with a 2-day difference between these subgroups; thus, 6 chickens from G3 and G4 were bled every other day. Blood was collected from the wing vein starting on the 4^th^ dpi through to the 28^th^ dpi. For hematology assays, all chickens from every group and subgroup had their blood (1 mL) collected with ethylenediaminetetraacetic acid as the anticoagulant. For serum biochemistry, three chickens from every group and subgroup had their blood (2 mL) collected without anticoagulant to obtain serum. Three independent experiments were performed; figure legends indicate the number of repetitions.

### Determination of parasitemia and temperature

Blood smears were obtained daily from a drop of blood taken from the nail of the bird; the sample was then Giemsa stained and observed under an Axioplan Zeiss microscope by bright field microscopy. Parasitemia was estimated as a percentage by counting the number of parasites in about 1000 erythrocytes found in 10 microscopic fields using an immersion objective (100×) lens [13]. Parasitemia of immature erythrocytes, monocytes, heterophils and thrombocytes was also estimated as a percentage by counting at least 100 cells in three individual blood smears. The birds’ temperatures in Celsius were evaluated daily between 3:00 and 4:00 pm by inserting a digital thermometer in the chickens’ cloaca.

### Classic hematology

Packed cell volume was determined by the microhematocrit technique. Hemoglobin concentration was determined by a colorimetric method following sample centrifugation (1600 × *g*, 5 min) before reading [36]. Blood was diluted (1:200) with phosphate buffer saline (PBS) and erythrocytes were counted in a Neubauer chamber. Mean corpuscular volume (MCV) and mean corpuscular hemoglobin concentration (MCHC) were calculated [37]. Thrombocytes and leukocytes were counted simultaneously in a Neubauer chamber by diluting (1:50) blood with PBS containing 0.01% brilliant blue cresyl [36]. Later the percentage of thrombocytes in relation to leukocytes was determined by counting 100 cells in a blood smear [38]. The differential count of leukocytes was made in blood smears stained with Giemsa; 100 cells were counted per slide. Cells, infected or not, were observed under an Axioplan Zeiss microscope equipped with polarized light and images were captured with a digital camera (Axiovision MRc5). The percentage of immature erythrocytes and toxic heterophils was estimated by counting 300 and 100 cells, respectively, in at least 3 individual blood smears of chickens with high parasitemia. The cell size of thrombocytes, lymphocytes and monocytes was estimated in control (noninfected), low (15%) and high (50%) parasitemia chickens (5 animals each). Images of cells from individual blood smears were captured with the digital camera. The area of at least 250 to 170 for thrombocytes, 240 to 50 for lymphocytes and 100 to 25 for monocytes was estimated using the Axiovision software.

### Serum biochemistry

In the laboratory, serum was aliquoted and stored at −70°C until the assays. Alanine aminotransferase (ALT), aspartate aminotransferase (AST), lactate dehydrogenase (LDH), alkaline phosphatase (ALP), cholesterol, urea, uric acid and creatinine were analyzed using standard kits (Labtest Diagnostica SA, Brazil) with the aid of a spectrophotometer (BTS 310, Biosystems).

### Plasma nitric oxide production

Production of NO in plasma was evaluated by the Griess reaction [39] after converting nitrate to nitrite by incubation of plasma in nitrate reductase (purified from *Aspergillus*) in the presence of NADPH [40]. Ethylenediaminetetraacetic acid was used as an anticoagulant to avoid precipitation that may occur when the Griess solution is added to heparinized plasma. The mixture was incubated for 3 h at room temperature and 100 μL of the sample was mixed with 100 μL of the Griess reagent on a 96-well plate. After 10 min, the samples were read using a spectrophotometer (540 nm). The concentrations of nitrite in the samples were determined by the use of a standard curve with serial dilutions of sodium nitrite solution, and values were expressed in μM.

### Culture and staining of thrombocytes

Chicken leukocytes were separated from total blood on a 60% Percoll solution, as previously reported [41]. Blood from chickens, infected or noninfected, treated or not with AG, were used. Briefly, 3 mL of blood was collected into heparinized syringes, diluted (1:1) with Dulbecco’s Modified Eagle’s Medium (DMEM) layered on top of the Percoll solution and centrifuged at 600 *g* for 20 min without a break. The diluted plasma was discarded, and the buffy coat collected and washed. Cells, consisting mainly of lymphocytes, monocytes and thrombocytes, were resuspended in DMEM, adjusted to 2 × 10^7^ cells/mL and seeded on glass coverslips (150 μL) in 24-well plates for tissue culture. After 1 h of culture (adherence time) at 37°C in a 5% CO_2_ atmosphere, non-adherent cells were washed out and DMEM containing 10% fetal bovine serum (FBS) was added for 24 h of culture. Some coverslips were removed, and the cells were fixed in 4% formaldehyde in PBS and processed for immunofluorescence (see below) or stained with Giemsa, dehydrated in acetone-xylol and mounted in Entellan. Morphological observations were carried out under an Axioplan Zeiss microscope.

### Immunofluorescence analysis of thrombocytes

After fixing, cell monolayers were washed with PBS and incubated with ammonium chloride (100 mM) in PBS for 30 min. Cells were further incubated with 1.5% bovine serum albumin (BSA) in PBS (PBS/BSA) and incubated for 1 h in primary antibody diluted 1:10 in PBS/BSA. The primary antibody (anti-thrombocyte 11C3) was a supernatant of hybridoma cultures and a kind gift from Dr Kanellopoulos-Langevin C. [42]. This monoclonal antibody recognizes the chicken integrin CD41/61, which is a heterodimeric cell surface protein expressed on chicken thrombocytes [42]. The cells were washed, incubated for 10 min in PBS/BSA, and further incubated with anti-mouse secondary antibody conjugated with TRITC diluted 1:100 in PBS/BSA. The cells were washed and mounted with ProlongGold® with DAPI. Slides were observed under an Axioplan Zeiss microscope equipped with fluorescence.

### Histopathology

Chickens, at the acute phase of the infection, were sacrificed; spleens and livers were dissected and fixed in 10% buffered formalin. Samples were routinely processed in alcohol and xylol and embedded in paraffin. Paraffin blocks were cut using a rotational microtome. Sections of 5 μm were stained with hematoxylin and eosin (H&E) and observed under an Axioplan Zeiss microscope.

### Data analyses

Values are expressed as means and standard deviation. The results are also shown by correlating survival or parasitemia with days; due to the great variability of hematological values, the results for NO serum levels, hematology and biochemical assays of chickens were grouped by parasitemia range. The number of chickens in each parasitemia range of AG treated and untreated chickens are given in the figure legends. Survival was presented as a Kaplan-Meyer graph with the Mantel-Cox test analysis. The AG treated and untreated chickens were analyzed statistically using the Wilcoxon test; for comparison of noninfected and infected chickens the ANOVA test followed by the Dunnet test were used. The threshold of significance was *p* ≤ 0.05 or *p* ≤ 0.01, as indicated in the figure legends. Comparisons of cell size between thrombocytes, lymphocytes and monocytes of noninfected, low or high parasitemia chickens were performed using the Kruskal–Wallis test (α = 0.05).

## Results

**Figure 1:**
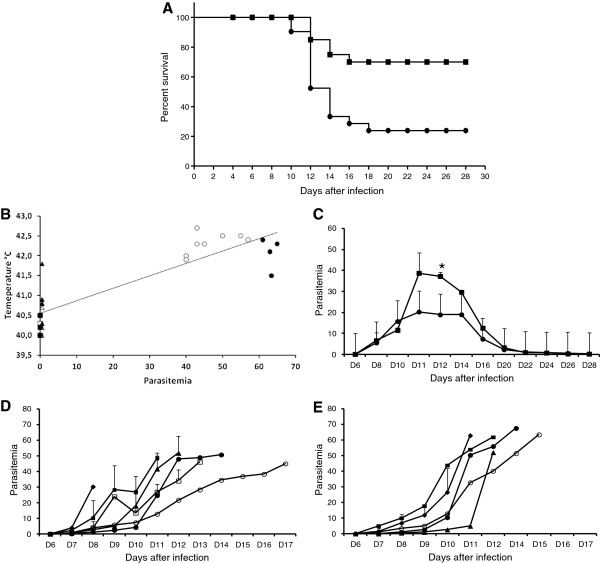
**Survival, temperature and parasitemia of chickens infected with *Plasmodium gallinaceum.*** (**A**) Survival of infected chickens treated (■) or not (●) with aminoguanidine. Mantel-Cox test *p* = 0.0021. (**B**) Correlation of temperature and parasitemia of noninfected, chronic and infected chickens treated (■,▲,●, respectively) or not (□, ∆, ○, respectively). (**C**) Parasitemia of chickens that chronified, when treated (■, *n* = 17) or not (●, *n* = 8). (**D**) Parasitemia of untreated chickens that died (♦, *n* = 1; ■, *n* = 6; ▲, *n* = 5; □, *n* = 2; ●, *n* = 1; ○, *n* = 1). (**E**) Parasitemia of treated chickens that died (♦, *n* = 2; ■, *n* = 1; ▲, *n* = 1; ●, *n* = 1; ○, *n* = 1). *Statistically significant (*p* < 0.05) in relation to the respective untreated value. Data are from two independent experiments, except “B” which is from one representative experiment.

Infected erythrocytes were found on day 6 dpi in AG-treated and untreated chickens. Only 33% of the AG-treated chickens died, while 66% of the untreated group succumbed during the experiment (Figure 1A). Typical clinical avian malaria manifestations, such as apathy, lack of appetite, cachexia, paleness of the cockscomb and legs, nystagmus, retracted body position with beak down and involuntary muscular face contraction were observed. These manifestations were less evident in chickens treated with AG. One animal from the untreated group presented abnormal rhythm vocalization. An additional movie file shows this in more detail (see Additional file 1). Body temperature raised (fever) similarly and on the day of death was equal in both chicken groups (Figure 1B). AG-treated chickens that chronified during the experiment presented a higher parasitemia peak (Figure 1C). Moreover, the AG-treated chickens presented an approximately 34% higher parasitemia on the day of death, in comparison to the untreated chickens (Figure 1D, E). Thus, treatment reduced clinical manifestations and increased survival but resulted in higher parasitemia.

**Figure 2:**
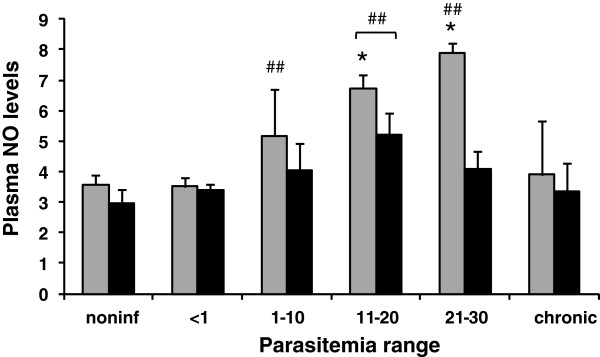
**Plasma nitric oxide levels (μM) of chickens infected with *Plasmodium gallinaceum, *treated (black bars) or not (gray bars), with aminoguanidine and grouped by parasitemia ranges (PR).** For noninfected chickens: *n* = 15 for untreated and *n* = 13 for treated; for PR of <1: *n* = 6 for untreated and n = 4 for treated; for PR of 1–10: *n* = 6 for untreated and *n* = 9 for treated; for PR of 11–20: *n* = 4 for untreated and treated; for PR of 21–30: *n* = 4 for untreated and treated; for chronic chickens: *n* = 18 for untreated and *n* = 10 for treated. ## Statistically significant (*p* < 0.01) in relation to the noninfected value. *Statistically significant (*p* < 0.05) in relation to the respective treated value. Data are from one representative experiment.

NO plasma levels did not change at low parasitemia (< 1) in chickens, whether treated or not with AG (Figure 2). However, plasma NO levels positively correlated with moderate and higher parasitemia in chickens not treated with AG (Figure 2). As expected, the administration of AG reduced NO in plasma (Figure 2). In animals with chronic infection, the NO plasma levels were similar to those of noninfected chickens, independently of AG treatment (Figure 2). Thus, systemic NO production was controlled by AG treatment.

**Figure 3:**
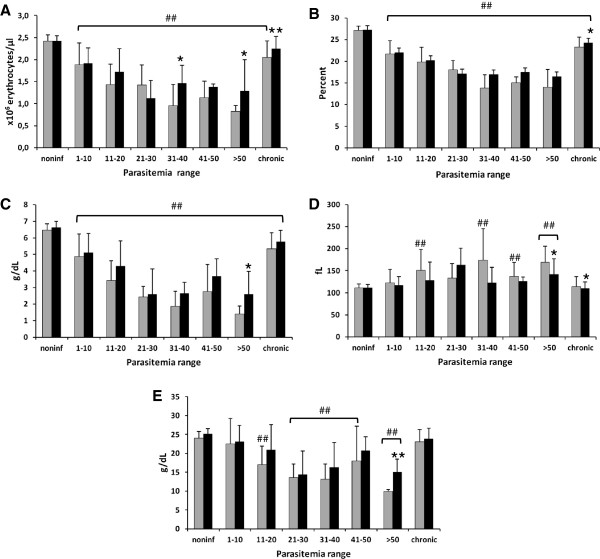
**Red cell hematological parameters of chickens infected with *Plasmodium gallinaceum, *treated (black bars) or not (gray bars), with aminoguanidine and grouped by parasitemia ranges (PR).** (**A**) Number of erythrocytes. (**B**) Hematocrit. (**C**) Hemoglobin concentration. (**D**) MCV. (**E**) MCHC. For noninfected chickens: *n* = 22 for untreated and treated; for PR of 1–10: *n* = 24 for untreated and *n* = 30 for treated; for PR of 11–20: *n* = 14 for untreated and *n* = 16 for treated; for PR of 21–30: *n* = 5 for untreated and *n* = 11 for treated; for PR of 31–40: *n* = 6 for untreated and *n* = 7 for treated; for PR of 41–50: *n* = 4 for untreated and *n* = 6 for treated; for PR of > 50: *n* = 4 for untreated and treated; for chronic chickens: *n* = 44 for untreated and *n* = 46 for treated. ## Statistically significant (*p* < 0.01) in relation to the noninfected value. *Statistically significant (*p* < 0.05 or *p* < 0.01 for **) in relation to the respective untreated value. Data are from two independent experiments.

Chickens were examined by classical hematology during the time course of the experiments. All three values of the red series reduced with higher parasitemia (Figure 3A-C). However, chickens treated with AG presented higher values, some of which were statistically significant, for the number of erythrocytes (Figure 3A) and hemoglobin concentration (Figure 3C), in relation to the untreated group. In addition, AG-treated chickens recovered their erythrocyte numbers when they became chronically infected (Figure 3A). The MCV increased with higher parasitemia for untreated chickens, while the AG-treated chickens had similar values in relation to noninfected chickens, except at the higher parasitemia range (Figure 3D). The MCHC reduced with higher parasitemia, but AG treatment increased these values in comparison to those of untreated chickens, especially at the higher parasitemia range (Figure 3E). In short, the AG treatment lowered the severity of the anemia caused by the infection.

**Figure 4:**
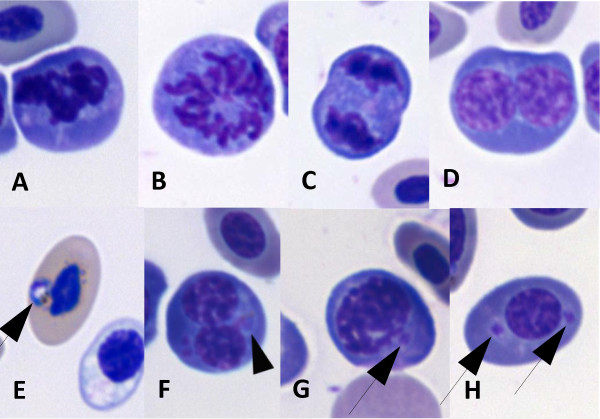
**Blood of chickens infected with *Plasmodium gallinaceum *presented immature erythrocytes that were also infected.** Erythrocyte at prophase (**A**), prometaphase (**B**), anaphase (**C**) and telophase (**D**) were seen. Mature (**E**) and immature erythrocytes (**F**, **G**, **H**) were infected (arrowheads).

**Figure 5:**
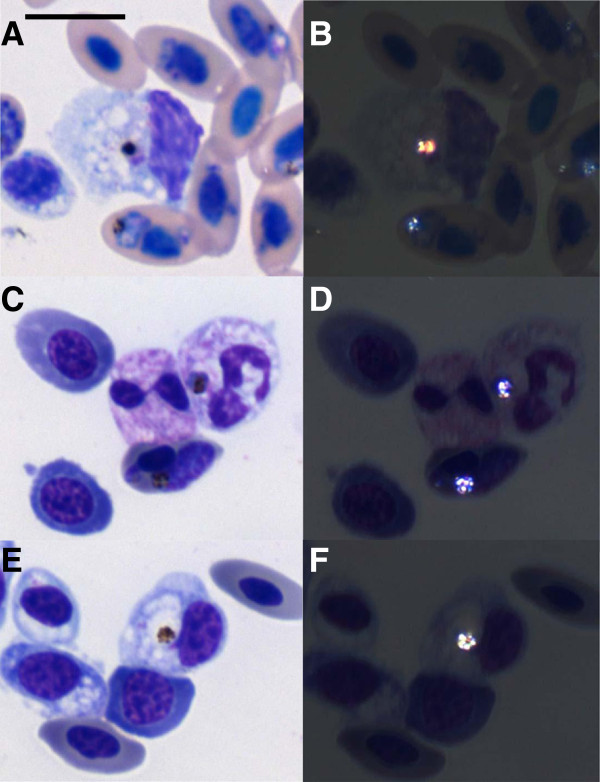
***Plasmodium gallinaceum *infects monocytes (A, B), heterophils (C, D) and thrombocytes (E, F).** (**A**, **C**, **E**) Leukocytes from blood smears stained with Giemsa. (**B**, **D**, **F**) Same visual field observed by polarized microscopy; note the birefringent malaria pigment. Bar = 10 μm.

Observations of peripheral blood smears of AG treated or untreated chickens with parasitemia above 35% revealed the presence of 19.2% of immature erythrocytes some undergoing the process of cell division (Figure 4A-D). Most of the infected erythrocytes were in a mature stage (Figure 4E), but 13.0% of immature erythrocytes in different phases of the cell cycle were also infected (Figure 4F-H). Some blood cells were also found infected in chickens with parasitemia above 40%: 1.0% of the monocytes (Figure 5A, B), 5.0% of heterophils (Figure 5C, D) and 4.2% of thrombocytes (Figure 5E, F). No differences were found between AG treated or untreated chickens.

**Figure 6:**
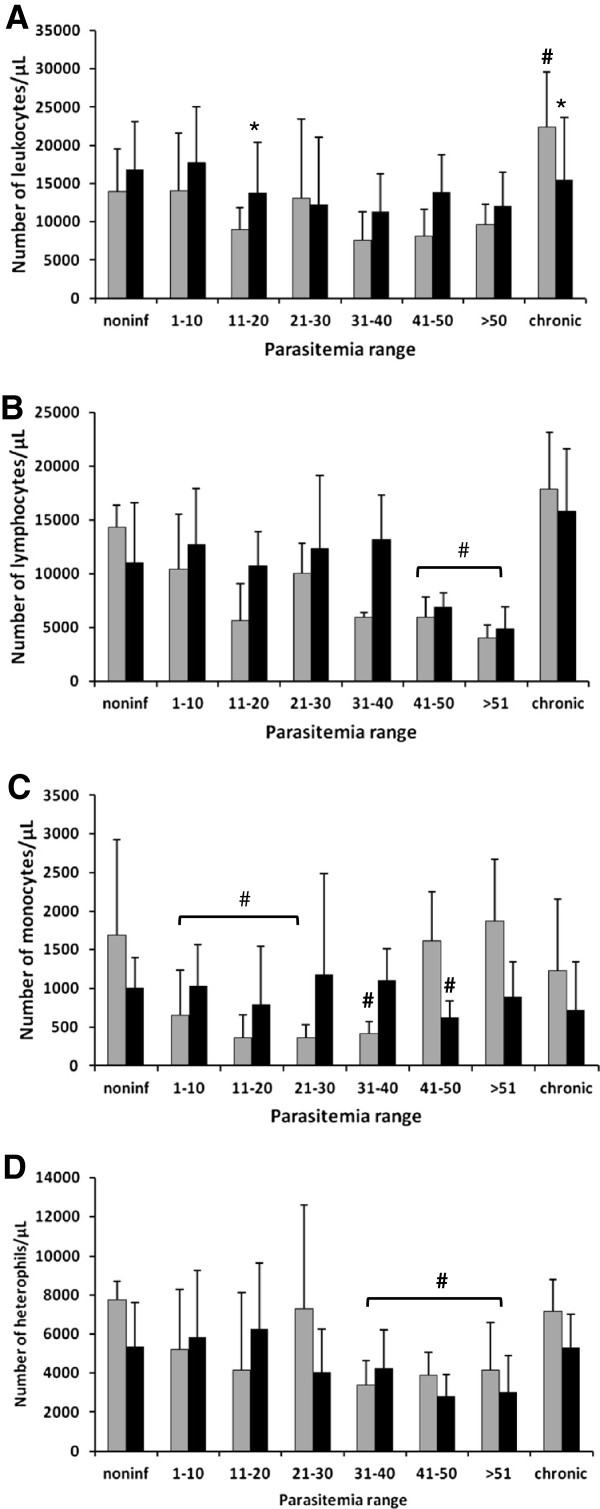
**Absolute numbers of total leukocytes (A), lymphocytes (B), monocytes (C) and heterophils (D) of chickens infected with *Plasmodium gallinaceum *treated (black bars) or not (gray bars) with aminoguanidine grouped by parasitemia range (PR).** For noninfected chickens: *n* = 11 for untreated and *n* = 12 for treated; for PR of 1–10: *n* = 17 for untreated and *n* = 19 for treated; for PR of 11–20: *n* = 8 for untreated and *n* = 16 for treated; for PR of 21–30: *n* = 3 for untreated and n = 6 for treated; for PR of 31–40: *n* = 5 for untreated and *n* = 5 for treated; for PR of 41–50: *n* = 4 for untreated and *n* = 4 for treated; for PR of >50: *n* = 3 for untreated and *n* = 4 for treated; for chronic chickens: *n* = 22 for untreated and *n* = 16 for treated. # Statistically significant (*p* < 0.05) in relation to the noninfected value; * Statistically significant (*p* < 0.05) in relation to the respective untreated value.

As parasitemia increased, AG treated and untreated chickens presented a trend towards leucopenia (Figure 6A). Treated chickens that survived the infection recovered the normal amount of leukocytes, while untreated chickens presented higher levels of leukocytes (Figure 6A). The most abundant leukocytes were lymphocytes, followed by heterophils and monocytes. A significant reduction in the total number of lymphocytes was detected in the two highest parasitemia ranges in both groups of chickens (Figure 6B). Monocytopenia was observed in both groups in the low parasitemia range (Figure 6C). At moderate parasitemia, monocytopenia was detected only in the untreated group (Figure 6C). Normal numbers of monocytes were registered in untreated chickens at the higher parasitemia range; however, treated chickens presented a lower number of monocytes (Figure 6C). A decrease in the number of heterophils was observed in both groups of chickens when higher parasitemia was achieved (Figure 6D). Chronified chickens tended to recover the number of these three leukocytes (Figure 6B-D). Eosinophils disappeared from the blood in chickens at moderate and higher parasitemia ranges (not shown).

**Figure 7:**
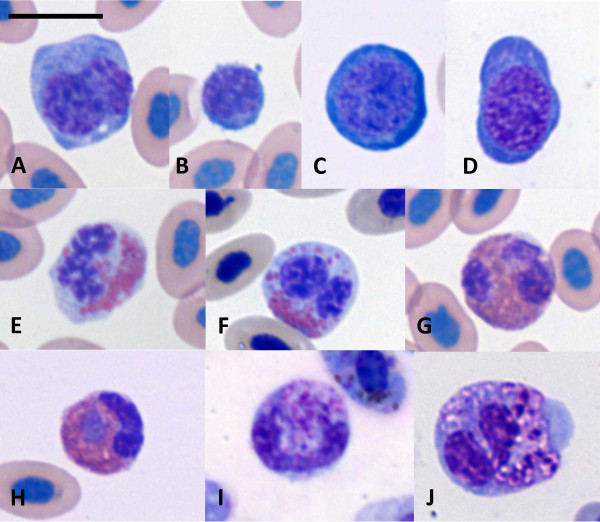
**Leukocytes from chickens, infected or noninfected with *Plasmodium gallinaceum.*** Monocyte (**A**) and lymphocytes (**B-D**) of noninfected (**A, B**) and infected chickens with low (**C**) or high parasitemia (**D**). Eosinophils from noninfected (**E**) and infected (**F**) chickens. Heterophils were normal in noninfected (**G**) and low parasitemia (**H**) animal; with higher parasitemia band (**I**) and toxic (**J**) heterophils were observed. Bar = 10 μm.

Blood lymphocytes and heterophils changed their morphology as parasitemia increased; however, monocytes and eosinophils did not (Figure 7). Monocytes of noninfected chickens presented purple, kidney-shaped nuclei and a light-blue cytoplasm with few deep-blue areas (Figure 7A). No statistical difference of monocyte size was found for noninfected, low or high parasitemia chickens (not shown). As expected, lymphocytes of noninfected chickens had a low cytoplasm nucleus ratio and were smaller in relation to monocytes (Figure 7B); their mean area was 57.4 μm^2^. Infection caused lymphocytes to increase in size with a tendency to lower the cytoplasm-nucleus ratio; irregular staining of the cytoplasm was more frequent (Figure 7C, D); their mean area was 72.1 μm^2^, statistically different from lymphocytes of noninfected chickens. Eosinophils were rarely observed and no apparent morphological change was seen when this cell type was compared between noninfected (Figure 7E) and low parasitemia infected chickens (Figure 7F). Heterophils of noninfected (Figure 7G) and moderately-infected chickens (Figure 7H) had similar morphology; but chickens with higher parasitemia presented normal (not shown), toxic band (Figure 7I) and toxic lobulated heterophils (Figure 7J). In chickens with high parasitemia, toxic heterophils represented 21.8% of the heterophil population. No clear changes in the morphology of these leukocytes were found during the infection between AG treated and untreated chickens. 

**Figure 8:**
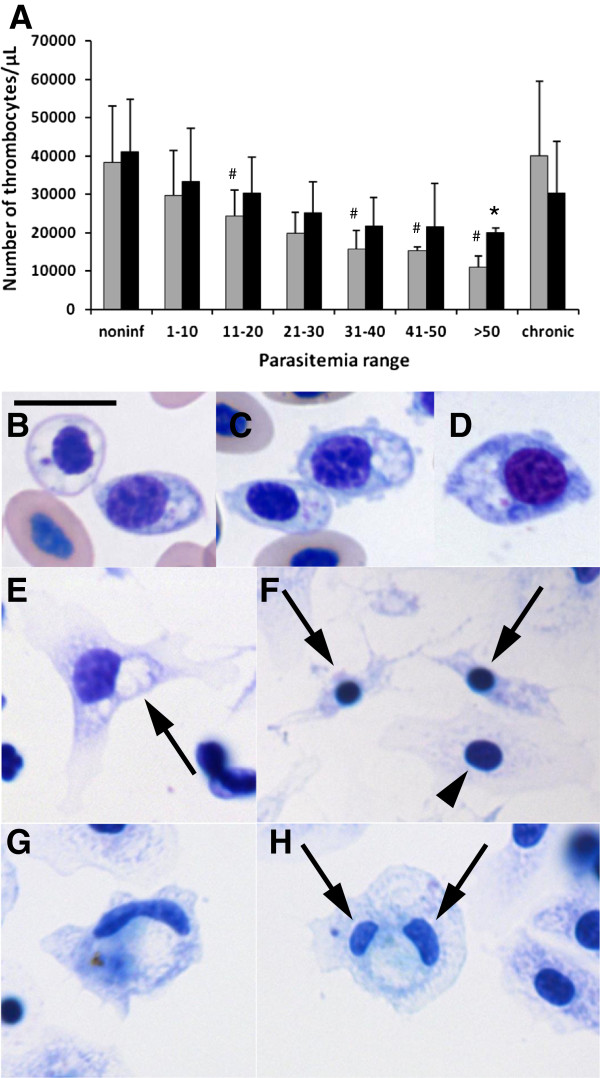
**Absolute numbers of thrombocytes (A) of chickens infected with *Plasmodium gallinaceum, *treated (black bars) or not (gray bars), with aminoguanidine and grouped by parasitemia range (PR).** Thrombocytes of infected chickens stained with Giemsa in blood smears (**B** - **D**) and after culture for 1 h (**E** - **H**)*.* Thrombocytes became larger and with more vacuoles when parasitemia was higher (**B** - **D**). (**E**) Normal thrombocytes with typical vacuoles (arrow). (**F**) Pyknotic thrombocytes. Note condensed chromatin (arrows). Atypical thrombocytes with long mononucleus (**G**) and two nuclei (arrows) (**H**) are seen. Bar = 10 μm. For the number of chickens by PR, number of experiments and significance of symbols, see Figure 6.

Untreated chickens had a significant thrombopenia that correlated to higher parasitemia, which was not seen in the AG treated chickens (Figure 8A). Thrombocytes also presented morphological changes with higher parasitemia, becoming larger with an increase in the cytoplasmic region where vacuoles are located (Figure 8B-D). The mean area of the thrombocytes was 30.9 μm^2^ for noninfected, 52.8 μm^2^ for low parasitemia, and 70.6 μm^2^ for higher parasitemia, all statistically significant. Thrombocytes were cultured to better characterize its morphology. Thrombocytes of noninfected chickens were cultured for 1 h, after which they presented a spread nucleus with patches of stained chromatin, as well as spread cytoplasm with evident vacuoles (Figure 8E). A few thrombocytes (< 4%) had a pyknotic nucleus with retracted cytoplasm (Figure 8F), indicating apoptosis, as reported before [65]. However, infection of the chickens induced the appearance of atypical thrombocytes with well spread cytoplasm with, either a long nucleus (Figure 8G) or two nuclei (Figure 8H).

**Figure 9:**
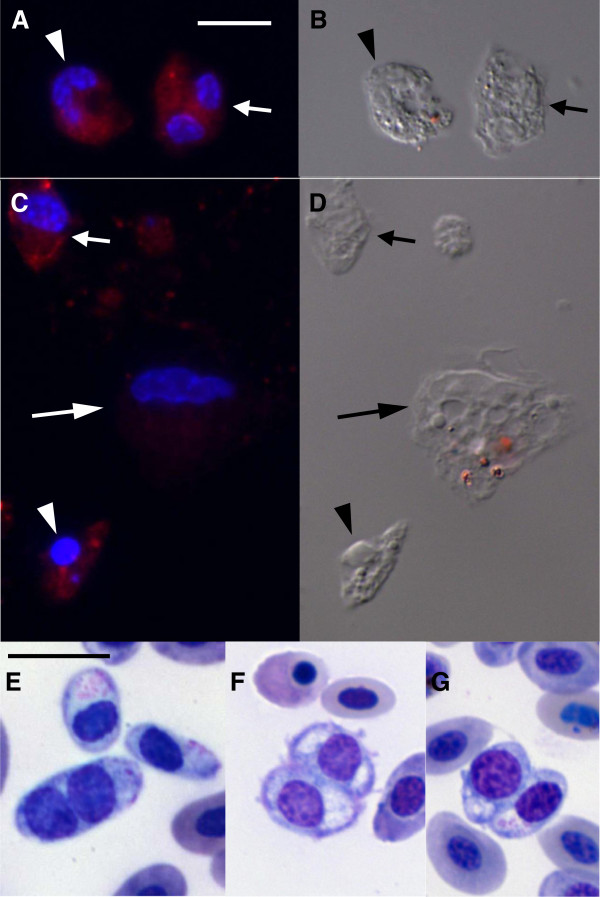
**Atypical thrombocytes were positive for the anti-thrombocyte antibody, 11C3, and were rarely detected in stained blood smears.** Fluorescence (**A**, **C**) and differential interference contrast (**B**, **D**) microscopy after labeling cells with the anti-thrombocyte antibody 11C3; red staining is the antibody labeling and blue is DNA. Cells were cultured for 1 (**A**, **B**) and 24 h (**C**, **D**). Bright field microcopy of stained blood smears (**E**, **F**, **G**). (**A**, **B**) Atypical thrombocytes with one nucleus (arrowhead) or two nuclei (arrow) cultured for 1 h were positive for 11C3. (**C**, **D**) Normal (arrow) and pyknotic (arrowhead) thrombocytes were positive for 11C3; macrophage (long arrow) was negative. (**E**- **G**) Atypical thrombocytes with two nuclei were also observed in blood smears of chickens with high parasitemia. Bar = 10 μm, bar in **A** and **E**, is the same for **B**, **C**, **D** and **F**, **G**, respectively.

These atypical cells were confirmed as thrombocytes by immunofluorescence (Figure 9). Cells with typical thrombocyte morphology, but with long or two nuclei (Figure 9A, B) were recognized by the 113C monoclonal antibody specific for chicken thrombocytes [42]. Normal and pyknotic thrombocytes were also positive for this antibody after 24 h in culture, but macrophages were negative (Figure 9C, D). Blood smears also revealed thrombocytes with double nuclei, but were rarely seen (Figure 9E-G).

**Figure 10:**
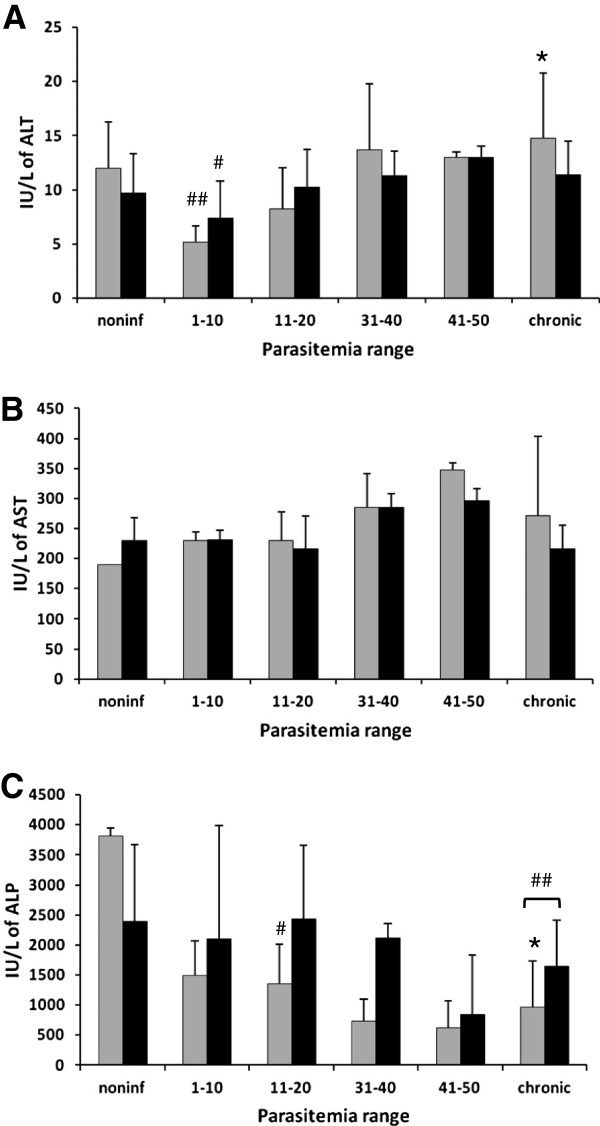
**Serum biochemistry of chickens infected with *Plasmodium gallinaceum, *treated (black bars) or not (gray bars) with aminoguanidine, and grouped by parasitemia ranges (PR).** (**A**) ALT, (**B**) AST, (**C**) ALP. For noninfected chickens, *n* = 6 for untreated and *n* = 7 for treated; for PR of 1–10, *n* = 7 for untreated and *n* = 6 for treated; for PR of 11–20, *n* = 6 for untreated and *n* = 4 for treated; for PR of 31–40, *n* = 6 for untreated and *n* = 5 for treated; for PR of 41–50, *n* = 5 for untreated and *n* = 4 for treated; for chronic chickens, *n* = 13 for untreated and *n* = 15 for treated. #Statistically significant (*p* < 0.05 or *p* < 0.01 for ##) in relation to the noninfected value; *Statistically significant (*p* < 0.05) in relation to the respective untreated value. Data are from two independent experiments.

Serum biochemistry revealed a reduction in the ALT levels in chickens of both groups in the low parasitemia ranges (Figure 10A). AST values increased in untreated chickens in the higher parasitemia range (Figure 10B). A reduction in ALP values was detected as parasitemia increased; chronic chickens also showed a reduced level of this enzyme and untreated animals presented a lower value (Figure 10C). Values for LDH, cholesterol, urea, and uric acid tended to increase with higher parasitemia for both chicken groups (not shown). No apparent changes in either chicken groups were detected for creatinine (not shown).

**Figure 11:**
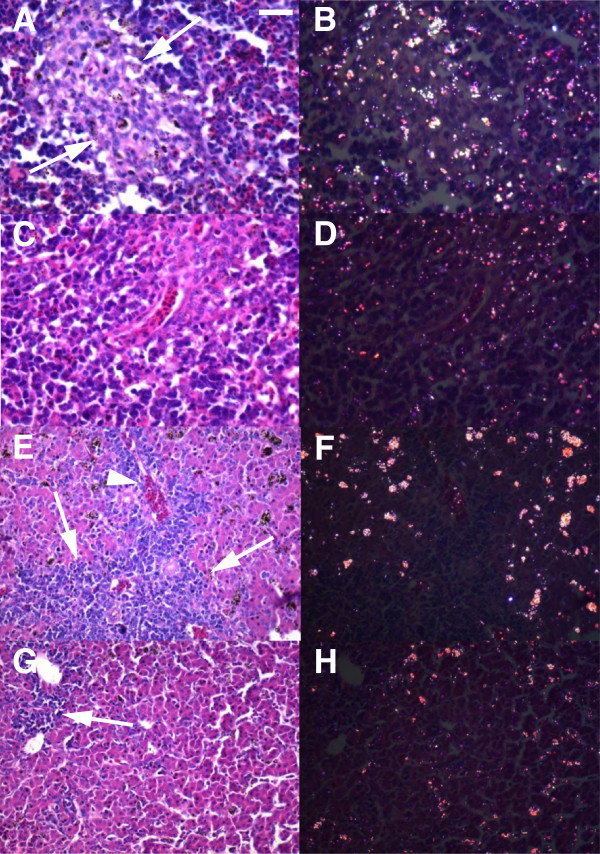
**Spleen and liver of aminoguanidine treated chickens have less malaria pigment during the acute phase of the infection.** (**A**) Spleen section of untreated chickens stained with H&E, showing lymphocyte depletion (arrows). (**B**) Same visual field observed by polarized microscopy. (**C**) Spleen section of treated chickens stained with H&E, showing extensive lymphocyte depletion. (**D**) Same visual field observed by polarized microscopy. Note less birefringent material in treated chickens. (**E**) Liver section of untreated chickens, stained with H&E. An extensive area of inflammatory infiltrate can be seen (arrows) next to a vessel (arrowhead). (**F**) Same visual field observed by polarized microscopy. (**G**) Liver section stained with H&E of treated chickens. A predominantly mixed mononuclear inflammatory infiltrate can be observed (arrow). (**H**) Same visual field observed by polarized microscopy. Note less birefringent material in treated chickens. Bar = 50 μm.

Spleens of noninfected chickens presented clear white and red pulp with normal tissue display (not shown). However, chickens with higher parasitemia presented spleen with lymphocyte depletion that was accentuated in AG treated chickens and moderate in untreated ones (Figure 11A, C). Treated chickens also presented less malaria pigment in the spleen (Figure 11B, D). Livers of untreated chickens with higher parasitemia presented a rich inflammatory infiltrate, composed of mononuclear cells and rare heterophils, with perivascular location (Figure 11E). Coagulation necrosis was also observed (not shown). AG treated chickens had scarce inflammatory infiltrate (Figure 11G) and less malaria pigment (Figure 11F, H) in the liver.

## Discussion

To better understand the role of NO in avian malaria, AG was used to pharmacologically block this radical previous to the infection of chickens with *P. gallinaceum*. AG treatment increased survival and reduced the levels of the clinical manifestation of chickens, when compared to the untreated group. Furthermore, treated chickens had higher parasitemia, indicating that survival is not directly related to parasitemia. Higher temperature (fever) was observed similarly between both groups. Infection caused anemia, but treatment with AG ameliorated the red series parameters. In addition, thrombocytopenia and inflammation, as seen by blood leukocyte morphology and tissue hemozoin, increased with infection, but were partially reverted by AG treatment. Collectively, these results indicate that AG treatment increased the survival of chickens infected with *P. gallinaceum* by partially reverting some of the pathological manifestations examined.

The higher parasitemia observed in AG treated chickens may be caused by a reduced capacity to uptake infected erythrocytes by spleen and liver macrophages that may extend the presence of infected erythrocytes in the blood. This is corroborated by lower levels of hemozoin in the spleen and liver of treated chickens. In addition, AG treatment caused lower phagocytic activity of rat macrophages [43], suggesting that the mechanism that increased erythrocyte in blood may be related to a reduced uptake capacity of chicken macrophages. The possibly lower uptake of infected erythrocytes by the spleen and liver of treated chickens may also explain the reduced levels of anemia found in these chickens. Another possible explanation for the higher parasitemia in AG-treated chickens may be the reduced capacity to control parasite growth. NO is a potential microbicidal agent [19,21,44,45] and blocking its production, as seen by the reduced plasma NO levels, indicates that AG caused an increase in the capacity of *P. gallinaceum* to persist in infected erythrocytes. In addition, less NO may cause parasites to increase in the entire tissue, resulting in a higher infection of erythrocytes.

The analysis of the red series parameters indicates that infection with *P. gallinaceum* induces a hypochromic macrocytic anemia. Although chickens of the AG group had higher parasitemia, they were less anemic. The MCV index, in particular, was reduced in treated chickens with higher parasitemia, when compared to untreated chickens. This difference in anemia can also be explained by a possibly lower uptake of infected erythrocytes by treated chickens, as discussed above, or lower lysis of these cells, caused by less NO. The observation of immature erythrocytes in the peripheral blood is a confirmation of the bone marrow’s response to the infection. *Plasmodium juxtanucleare*, another avian plasmodium, infects only mature erythrocytes [46,47], rarely reaching higher parasitemia levels [12]. The higher aggressiveness of *P. gallinaceum,* in relation to *P. juxtanucleare,* may also involve the capacity of the former to infect erythrocytes in all states of maturation.

*Plasmodium gallinaceum* also infects monocytes, heterophils and thrombocytes. Monocytes and heterophils are professional phagocytes [48,49] and may actively up take free parasites or hemozoin in the blood after lysis of erythrocytes. This has been seen in the blood smears of human subjects, in monocytes and neutrophils [27,50]. However, thrombocytes are not professional phagocytes [41] and the presence of parasites in these cells indicates the entrance of *P. gallinaceum* by active invasion.

A tendency to reduce the number of total leukocytes was seen with the infection of the chickens. However, a significant reduction of lymphocytes and heterophils was observed with the increase in parasitemia. Thus, the infection by *P. gallinaceum* reduced the number of some blood leukocytes. Children infected with *P. falciparum* have low lymphocyte counts due to spleen sequestration [27]. Thus, the leukopenia seen in *P. gallinaceum*-infected chickens may also be caused by tissue sequestration, especially of lymphocytes and heterophils. Monocytes displayed a different behavior probably explained by tissue sequestration at the beginning of the infection and faster recovery in relation to the other two leukocytes. This recovery may be related to hemozoin accumulation in tissue that induces monocyte chemokine secretion [51]. As hemozoin accumulates in the tissues of chickens, higher levels of chemokines may attract monocytes to the tissues and also increase the production of these cells in the bone marrow, compensating quantities in the blood. A similar response may occur in treated chickens but with a shorter time course. Further studies are necessary to better understand the fluctuation in the number of monocytes observed in both chicken groups. Another interesting result was the recovery of the number of leukocytes in chickens that became chronic, especially in the untreated group, which presented leukocytosis in relation to noninfected chickens. Data indicate that the inflammatory response in untreated chickens was elevated, resulting in high numbers of leukocytes in the blood during chronification.

Infection caused lymphocytes to increase in size and heterophils became toxic. The morphological changes of lymphocytes and heterophils with the infection indicate that these cells also became activated during the infection. These changes show that the immune system of chickens was generally activated by the infection, as suggested before [13]. Eosinophils make an important contribution to inflammation and the immune response; however, the function of avian eosinophils is not very well known [52,53]. It has been suggested that avian eosinophils may be involved in the early stage of acute inflammation [54]. It has also been demonstrated that eosinophils participate in the rat liver granuloma induced by *P. berghei*[55]. Children infected with malaria, however, have lower eosinophil numbers [27]. Eosinophils disappeared from the blood in the chicken malaria model, studied herein, indicating that the *P. gallinaceum* infection of chickens negatively modulates eosinophils right at the beginning of the infection process, indicating that this cell type is not involved in the pathogenesis of this disease.

As for the other malaria models [56,57], thrombocytopenia was also seen after infection of the chickens. Interestingly, AG treatment reduced thrombocytopenia; no significant difference in total thrombocyte number was found when compared to noninfected chickens. The reduction in the number of thrombocytes in untreated chickens may be related to the greater inflammation caused by the infection, which may induce a higher consumption of thrombocytes. Moreover, in severe human malaria, vasculitis [58,59] and disseminated intravascular coagulation [60,61] are pathological manifestations that consume platelets and may also occur in infected chickens. Another effect of AG may be related to the reduced vasodilation effect in chickens [35]. This effect would reduce tissue edema, which is a seminal characteristic of inflammation [62]. Less edema maintains the perfusion of tissues, which would help the animal to better survive the infection. AG treatment may ameliorate vasodilation, vasculitis and disseminated intravascular coagulation, explaining the lower thrombocytopenia. This thrombocytopenia, together with the lower anemia and lower general inflammation, may explain the lower death rate observed in treated chickens.

In addition, the infection caused an increase in thrombocyte size, and in the appearance of thrombocytes with long and double nuclei. These atypical cells were confirmed as thrombocytes by immunofluorescence. Culture helped in the identification of the atypical thrombocytes, as these cells spread completely over a substrate [63]. After observing these atypical thrombocytes, the blood smears were also re-examined and thrombocytes with double nuclei were also seen. The long and double nuclei may be evidence of immature thrombocytes being released into the blood, due to the consumption of these cells by the infection. As far as we know, this is the first description of atypical thrombocytes present in blood in chickens.

Serum biochemistry revealed few changes between the chicken groups and the chicken with higher parasitemia. Higher AST, lower ALP and the tendency to increase urea and uric acid with the infection suggest that the liver and the kidney were affected by the infection [64].

In conclusion, anemia, thrombocytopenia and inflammation are factors that result in host death in avian malaria. The administration of AG blocks NO, leading to a higher survival rate of the chickens and higher parasitemia. The improved survival of AG treated chickens may be due to lower inflammation, anemia and thrombocytopenia. The study of NO is important for furthering the understanding of the pathophysiology of malaria and inhibition of NO signaling may be an extra therapeutic approach for severe malaria.

## Abbreviations

NO: Nitric oxide; AG: Aminoguanidine; Dpi: Day post-infection; PBS: Phosphate buffer saline; MCV: Mean corpuscular volume; MCHC: Mean corpuscular hemoglobin concentration; ALT: Alanine aminotransferase; AST: Aspartate aminotransferase; LDH: Lactate dehydrogenase; ALP: Alkaline phosphatase; DMEM: Dulbecco’s Modified Eagle’s Medium; FBS: Fetal bovine serum; BSA: Bovine serum albumin.

## Competing interests

The authors declare that have no competing interests.

## Authors’ contributions

BMM participated in chicken maintenance, infection, treatment and necropsy, hematological, immunofluorescence and histological data acquisition, thrombocyte cultivation, statistical analysis and helped to draft the manuscript. FJBM participated in chicken maintenance, infection, treatment and necropsy, hematological, biochemical and histological data acquisition, thrombocyte cultivation, statistical analysis and helped to draft the manuscript. FSS participated in chicken maintenance and data acquisition and analysis of thrombocyte cultivation. ECQC participated in histological data acquisition and analysis and helped to draft the manuscript. APA participated in hematological and biochemical data acquisition and analysis. JLMN conceived of the study, participated in its design, and helped to draft the manuscript. RAD conceived of the study, participated in its design, participated in immunofluorescence data analysis, and helped to draft the manuscript. All authors read and approved the final manuscript.

## Supplementary Material

Additional file 1 **Abnormal rhythm vocalization of chickens infected with *Plasmodium gallinaceum. ***The video shows a chicken from the untreated group with a clear abnormal rhythm vocalization (Additional file 1). Click here for file

## References

[B1] WHO World Health Organization 2012 http://www.who.int/malaria/en/

[B2] John CC Kutamba E Mugarura K Opoka RO Adjunctive therapy for cerebral malaria and other severe forms of *Plasmodium falciparum* malaria Expert Rev Anti Infect Ther 2010 8 997 1008 10.1586/eri.10.90 20818944 PMC2987235

[B3] De Souza JB Riley EM Cerebral Malaria: the contribution of studies in animal models to our understanding of immunopathogenesis Microbes Infect 2002 4 291 300 10.1016/S1286-4579(02)01541-1 11909739

[B4] Slater LB Malarial birds: modeling infectious human disease in animals Bull Hist Med 2005 79 261 294 10.1353/bhm.2005.0092 15965289

[B5] Paraense WL Ações patogênicas das formas exo-eritrocitárias do *Plasmodium gallinaceum* Mem Inst Oswaldo Cruz 1946 44 147 191 In Portuguese 10.1590/S0074-02761946000100005

[B6] Anderson CR Continuous propagation of *Plasmodium gallinaceum* in chicken erythrocytes Am J Trop Med Hyg 1953 2 234 242 13040657 10.4269/ajtmh.1953.2.234

[B7] Garnham PCC Malaria parasites and other Haemosporidia 1966 Oxford, UK: Blackwell Scientific Publications 1 114

[B8] Williams RB The efficacy of a mixture of trimethoprim and sulphaquinoxaline against *Plasmodium gallinaceum* malaria in the domesticated fowl *Gallus gallus* Vet Parasitol 2005 129 193 207 10.1016/j.vetpar.2005.01.011 15845274

[B9] Krettli AU Andrade-Neto VF Brandão MG Ferrari WM The search for new antimalarial drugs from plants used to treat fever and malaria or plants randomly selected: a review Mem Inst Oswaldo Cruz 2001 96 1033 1042 10.1590/S0074-02762001000800002 11784919

[B10] Permin A Juhl J The development of *Plasmodium gallinaceum* infections in chickens following single infections with three different dose levels Vet Parasitol 2002 105 1 10 10.1016/S0304-4017(01)00645-8 11879962

[B11] Frevert U Späth GF Yee H Exoerythrocytic development of *Plasmodium gallinaceum* in the white leghorn chicken Int J Parasitol 2008 38 655 672 10.1016/j.ijpara.2007.09.012 18005972 PMC2430052

[B12] Silveira P DaMatta RA Dagosto M Hematological changes of chickens experimentally infected with *Plasmodium (Bennettinia)* juxtanucleare Vet Parasitol 2009 162 257 262 10.1016/j.vetpar.2009.03.013 19345020

[B13] Macchi BM Quaresma JAS Herculano AM Crespo-López ME DaMatta RA Do Nascimento JLM Pathogenic action of *Plasmodium gallinaceum* in chickens: brain histology and nitric oxide production by blood monocyte-derived macrophages Vet Parasitol 2010 172 16 22 10.1016/j.vetpar.2010.04.032 20537466

[B14] Braga EM Silveira P Belo NO Valkiūnas G Recent advances in the study of avian malaria: an overview with an emphasis on the distribution of *Plasmodium* spp in Brazil Mem Inst Oswaldo Cruz 2011 106 Suppl 1 3 11 21881752 10.1590/s0074-02762011000900002

[B15] Ghigo D Todde R Ginsburg H Costamagna C Gautret P Bussolino F Ulliers D Giribaldi G Deharo E Gabrielli G Pescarmona G Bosia A Erythrocyte stages of *Plasmodium falciparum* exhibit a high nitric oxide synthase (NOS) activity and release an NOS-inducing soluble factor J Exp Med 1995 182 677 688 10.1084/jem.182.3.677 7544394 PMC2192170

[B16] Favre N Ryffel B Rudin W Parasite killing in murine malaria does not require nitric oxide production Parasitology 1999 118 139 143 10.1017/S0031182098003618 10028527

[B17] Maneerat Y Viriyavejakul P Punpoowong B Jones M Wilairatana P Pongponratn E Turner GD Udomsangpetch R Inducible nitric oxide synthase expression is increased in the brain in fatal cerebral malaria Histopathology 2000 37 269 277 10.1046/j.1365-2559.2000.00989.x 10971704

[B18] Taylor-Robinson AW Phillips RS Severn A Moncada S Liew FY The role of TH1 and TH2 cells in a rodent malaria infection Science 1993 260 1931 1934 10.1126/science.8100366 8100366

[B19] Taylor-Robinson AW Antimalarial activity of nitric oxide: cytostasis and cytotoxicity towards *Plasmodium falciparum* Biochem Soc Trans 1997 25 262S 9191306 10.1042/bst025262s

[B20] Balmer P Phillips HM Maestre AE McMonagle FA Phillips RS The effect of nitric oxide on the growth of *Plasmodium falciparum*, *P. chabaudi* and *P. berghei* in vitro Parasite Immunol 2000 22 97 106 10.1046/j.1365-3024.2000.00281.x 10652122

[B21] Green SJ Nacy CA Antimicrobial and immunopathologic effects of cytokine-induced nitric oxide synthesis Curr Opin Infect Dis 1993 6 384 396

[B22] Mellouk S Offman SL Liu Z de la Vega P Billiar TR Nussler AK Nitric oxide-mediated antiplasmodial activity in human and murine hepatocytes induced by gamma interferon and the parasite itself: enhancement by exogenous tetrahydrobiopterin Infect Immun 1994 62 4043 4046 8063424 10.1128/iai.62.9.4043-4046.1994 PMC303065

[B23] Seguin MC Klotz FW Schneider I Weir JP Goodbary M Slayter M Raney JJ Aniagolu JU Green SJ Induction of nitric oxide synthase protects against malaria in mice exposed to irradiated *Plasmodium berghei* infected mosquitoes: involvement of interferon gamma and CD8^+^ T cells J Exp Med 1994 180 353 358 10.1084/jem.180.1.353 7516412 PMC2191552

[B24] Taylor-Robinson AW Smith EC A dichotomous role for nitric oxide in protection against blood stage malaria infection Immunol Lett 1999 67 1 9 10.1016/S0165-2478(98)00148-5 10217199

[B25] Casals-Pascual C Kai O Newton CR Peshu N Roberts DJ Thrombocytopenia in falciparum malaria is associated with high concentrations of IL-10 Am J Trop Med Hyg 2006 75 434 436 16968917

[B26] Cox D McConkey S The role of platelets in the pathogenesis of cerebral malaria Cell Mol Life Sci 2010 67 557 568 10.1007/s00018-009-0211-3 20091081 PMC11115904

[B27] Maina RN Walsh D Gaddy C Hongo G Waitumbi J Otieno L Jones D Ogutu BR Impact of *Plasmodium falciparum* infection on haematological parameters in children living in Western Kenya Malar J 2010 9 Suppl 3 S4 10.1186/1475-2875-9-S3-S4 21144084 PMC3002140

[B28] Gramaglia I Sahlin H Nolan JP Frangos JA Intaglietta M van der Heyde HC Cell- rather than antibody-mediated immunity leads to the development of profound thrombocytopenia during experimental *Plasmodium berghei* malaria J Immunol 2005 175 7699 7707 16301680 10.4049/jimmunol.175.11.7699

[B29] Togbe D Schofield L Grau GE Schnyder B Boissay V Charron S Rose S Beutler B Quesniaux VF Ryffel B Murine cerebral malaria development is independent of toll-like receptor signaling Am J Pathol 2007 170 1640 1648 10.2353/ajpath.2007.060889 17456769 PMC1854958

[B30] Faille D Combes V Mitchell AJ Fontaine A Juhan-Vague I Alessi MC Chimini G Fusaï T Grau GE Platelet microparticles: a new player in malaria parasite cytoadherence to human brain endothelium FASEB J 2009 23 3449 3458 10.1096/fj.09-135822 19535685

[B31] Bridges DJ Bunn J van Mourik JA Grau G Preston RJS Molyneux M Combes V O’Donnell JS Laat B Craig A Rapid activation of endothelial cells enables P. falciparum adhesion to platelet decorated von Willebrand factor strings Blood 2010 115 1472 1474 10.1182/blood-2009-07-235150 19897581 PMC2840836

[B32] Greenbaum DC FitzGerald GA Platelets, pyrexia and plasmodia New Engl J Med 2009 361 526 528 10.1056/NEJMcibr0903050 19641212

[B33] Allen PC Nitric oxide production during *Eimeria tenella* infections in chickens Poult Sci 1997 76 810 813 9181612 10.1093/ps/76.6.810

[B34] Wideman RF Erf GF Chapman ME Nω-Nitro-L-Arginine Methyl Ester (L-NAME) amplifies the pulmonary hypertensive response to microparticle injections in broilers Poultry Sci 2005 84 1077 1091 16050125 10.1093/ps/84.7.1077

[B35] Wideman RF Bowen OT Erf GF Chapman ME Influence of aminoguanidine, an inhibitor of inducible nitric oxide synthase, on the pulmonary hypertensive response to microparticle injections in broilers Poultry Sci 2006 85 511 527 16553284 10.1093/ps/85.3.511

[B36] Campbell TW Hematology of birds. In Veterinary Hematology and Clinical Chemistry 2004 Philadelphia, USA: Lippincott Williams & Wilkins 225 258

[B37] Jain NC Essentials of hematology 1993 Philadelphia, USA: Lea & Febiger

[B38] Silva WB Machado C Goldberg DW Moreira SB Silva LCCP Freire IMA Maciel PO Almosny NRP Assessment of blood inflammatory response in BCG stimulated rattlesnakes (*Crotalus durissus* Linnaeus, 1758) Pesq Vet Bras 2009 29 985 992 In Portuguese 10.1590/S0100-736X2009001200006

[B39] Green LC Wagner DA Skipper PL Analyses of nitrate, nitrite and nitrite in biological fluids Anal Biochem 1982 126 131 138 10.1016/0003-2697(82)90118-X 7181105

[B40] Haddad EK Duclos AJ Baines MG Early embryo loss is associated with local production of nitric oxide by decidual mononuclear cells J Exp Med 1995 182 1143 1152 10.1084/jem.182.4.1143 7561687 PMC2192282

[B41] DaMatta RA Seabra SH de Souza W Further studies on the phagocytic capacity of chicken thrombocytes J Submicrosc Cytol Pathol 1998 30 271 277 9648290

[B42] Lacoste-Eleaume AS Bleux C Quéré P Coudert F Corbel C Kanellopoulos-Langevin C Biochemical and functional characterization of an avian homolog of the integrin GPIIb-IIIa present on chicken thrombocytes Exp Cell Res 1994 213 198 209 10.1006/excr.1994.1191 8020592

[B43] Tümer C Bilgin HM Obay BD Diken H Atmaca M Kelle M Effect of nitric oxide on phagocytic activity of lipopolysaccharide-induced macrophages: possible role of exogenous L-arginine Cell Biol Int 2007 31 565 569 10.1016/j.cellbi.2006.11.029 17241792

[B44] Guillermo LVC DaMatta RA Nitric oxide inhibition after *Toxoplasma gondii* infection of chicken macrophage cell lines Poult Sci 2004 83 776 782 15141835 10.1093/ps/83.5.776

[B45] Koka S Lang C Niemoeller OM Boini KM Nicolay JP Huber SM Lang F Influence of NO synthase inhibitor L-NAME on parasitemia and survival of *Plasmodium berghei* infected mice Cell Physiol Biochem 2008 21 481 488 10.1159/000129641 18453756

[B46] Bennet FG Warrem M Cheong WH Biology of the Malaysian strain of Plasmodium juxtanucleare Versiani and Gomes, 1941. II. The sporogonic stages in Culex (culex) sitiens Wiedmann J Parasitol 1966 52 647 652 10.2307/3276421 5969102

[B47] Krettli AU *Plasmodium juxtanucleare* in the state of Minas Gerais, Brazil. Studies on its prevalence and some aspects of its biology Rev Inst Med Trop 1972 14 235 245 5084218

[B48] Stabler JG McCormick TW Powell KC Kogut MH Avian heterophils and monocytes: phagocytic and bactericidal activities against *Salmonella enteritidis* Vet Microbiol 1994 38 293 305 10.1016/0378-1135(94)90148-1 8160345

[B49] van Dijk A Tersteeg-Zijderveld MHG Tjeerdsma-van Bokhoven JLM Jansman AJM Veldhuizen EJA Haagsman HP Chicken heterophils are recruited to the site of Salmonella infection and release antibacterial mature Cathelicidin-2 upon stimulation with LPS Mol Immunol 2009 46 1517 1526 10.1016/j.molimm.2008.12.015 19187966

[B50] Abdalla SH Peripheral blood and bone marrow leucocytes in Gambian children with malaria: numerical changes and evaluation of phagocytosis Ann Trop Paediatr 1988 8 250 258 2467614 10.1080/02724936.1988.11748582

[B51] Jaramillo M Plante I Ouellet Vandal K Tessier PA Olivier M Hemozoin-inducible proinflammatory events in vivo: potential role in malaria infection J Immunol 2004 172 3101 3110 14978116 10.4049/jimmunol.172.5.3101

[B52] Maxwell MH The avian eosinophil: a review World Poultry Sci J 1987 3 190 207

[B53] de Andrade JG de Carvalho EC de Santos CP DaMatta RA Mixed infection with *Libyostrongylus dentatus* and *Libyostrongylus douglassii* induces a heterophilic inflammatory infiltrate in the proventriculus of ostriches Avian Pathol 2011 40 367 370 10.1080/03079457.2011.585631 21812715

[B54] Barnes HJ Fletcher OJ Aziz TA Fletcher OJ, Aziz TA Hemic system Avian Histopathology 2008 3 Jacksonville, FL: AAAP 1 23

[B55] Khan ZM Vanderberg JP Eosinophil-rich, granulomatous inflammatory response to *Plasmodium berghei* hepatic schizonts in nonimmunized rats is age-related Am J Trop Med Hyg 1991 45 190 201 1877714 10.4269/ajtmh.1991.45.190

[B56] Piguet PF Kan CD Vesin C Thrombocytopenia in an animal model of malaria is associated with an increased caspase-mediated death of thrombocytes Apoptosis 2002 7 91 98 10.1023/A:1014341611412 11865192

[B57] Jadhav UM Patkar VS Kadam NN Thrombocytopenia in malaria - correlation with type and severity of malaria J Assoc Physicians India 2004 52 615 618 15847353

[B58] Gopinathan VP Bhalla IP Peripheral vasculitis associated with falciparum malaria J Assoc Physicians India 1987 35 742 743 3328749

[B59] Patel DN Pradeep P Surti MM Agarwal SB Clinical manifestations of complicated malaria – an overview JIACM 2003 4 323 331

[B60] Moreno A García A Cabrera-Mora M Strobert E Galinski MR Case report: disseminated intravascular coagulation complicated by peripheral gangrene in a *Rhesus* macaque (*Macaca mulatta*) experimentally infected with *Plasmodium coatneyi* Am J Trop Med Hyg 2007 76 648 654 17426164

[B61] Tessier-Marteau A Cruguel S Grand F Asfar P Zandecki M Macchi L DIC and peripheral gangrene in a severe Plasmodium falciparum malaria: the coagulation-inflammation cycle with *Plasmodium falciparum* as a model Ann Biol Clin (Paris) 2009 67 569 572 19789130 10.1684/abc.2009.0367

[B62] Renkin EM Cellular aspects of transvascular exchange: a 40-year perspective Microcirculation 1994 1 157 167 10.3109/10739689409148270 8790586

[B63] DaMatta RA Manhães L Lassounskaia E De Souza W Chicken thrombocytes in culture: lymphocyte-conditioned medium delays apoptosis Tissue Cell 1999 31 255 263 10.1054/tice.1999.0002 10481297

[B64] Misra DP Das S Pattnaik M Singh SC Jena RK Relationship of hepatic and renal dysfunction with haemorrheological parameters in *Plasmodium falciparum* malaria J Assoc Physicians India 2011 59 552 556 22334967

